# SARS-CoV-2 ORF6 protein does not antagonize interferon signaling in respiratory epithelial Calu-3 cells during infection

**DOI:** 10.1128/mbio.01194-23

**Published:** 2023-06-28

**Authors:** Minghua Li, Kasirajan Ayyanathan, Mark Dittmar, Jesse Miller, Iulia Tapescu, Jae Seung Lee, Marisa E. McGrath, Yong Xue, Sanjay Vashee, David C. Schultz, Matthew B. Frieman, Sara Cherry

**Affiliations:** 1 Department of Pathology, University of Texas Medical Branch, Galveston, Texas, USA; 2 Department of Pathology and Laboratory Medicine, University of Pennsylvania, Philadelphia, Pennsylvania, USA; 3 Department of Microbiology and Immunology, Center for Pathogen Research, University of Maryland School of Medicine, Baltimore, Maryland, USA; 4 J Craig Venter Institute, Rockville, Maryland, USA; 5 Department of Biochemistry and Biophysics, University of Pennsylvania, Philadelphia, Pennsylvania, USA; Icahn School of Medicine at Mount Sinai, New York, New York, USA

**Keywords:** SARS-CoV-2, ORF6, interferon, antagonism

## Abstract

Severe acute respiratory syndrome coronavirus 2 (SARS-CoV-2) has caused millions of deaths, posing a substantial threat to global public health. Viruses evolve different strategies to antagonize or evade host immune responses. While ectopic expression of SARS-CoV-2 accessory protein ORF6 blocks interferon (IFN) production and downstream IFN signaling, the role of ORF6 in IFN signaling during *bona fide* viral infection of respiratory cells is unclear. By comparing wild-type (WT) and ORF6-deleted (ΔORF6) SARS-CoV-2 infection and IFN signaling in respiratory cells, we found that ΔORF6 SARS-CoV-2 replicates more efficiently than WT virus and, thus, stimulates more robust immune signaling. Loss of ORF6 does not alter innate signaling in infected cells: both WT and ΔORF6 virus induce delayed IFN responses only in bystander cells. Moreover, expression of ORF6 in the context of SARS-CoV-2 infection has no effect on Sendai virus-stimulated IFN induction: robust translocation of IRF3 is observed in both SARS-CoV-2 infected and bystander cells. Furthermore, IFN pretreatment potently blocks WT and ΔORF6 virus replication similarly, and both viruses fail to suppress the induction of interferon-stimulated genes (ISGs) upon IFN-β treatment. However, upon treatment with IFN-β, only bystander cells induce STAT1 translocation during infection with WT virus, whereas ΔORF6 virus-infected cells now show translocation. This suggests that under conditions of high IFN activation, ORF6 can attenuate STAT1 activation. These data provide evidence that ORF6 is not sufficient to antagonize IFN production or IFN signaling in SARS-CoV-2-infected respiratory cells but may impact the efficacy of therapeutics that stimulate innate immune pathways.

**IMPORTANCE:**

Previous studies identified several SARS-CoV-2 proteins, including ORF6, that antagonize host innate immune responses in the context of overexpression of viral proteins in non-respiratory cells. We set out to determine the role of ORF6 in IFN responses during SARS-CoV-2 infection of respiratory cells. Using a deletion strain, we observed no reduction of infection and no difference in evasion of IFN signaling, with responses limited to bystander cells. Moreover, stimulation of Sendai virus-induced IFN production or IFN-β-stimulated ISG expression was comparable between SARS-CoV-2 virus and SARS-CoV-2 lacking ORF6 virus, suggesting that ORF6 is not sufficient to counteract IFN induction or IFN signaling during viral infection.

## INTRODUCTION

Severe acute respiratory syndrome coronavirus 2 (SARS-CoV-2) is a recently emerged coronavirus that causes coronavirus disease 2019 (COVID-19) and a global pandemic (1, 2). SARS-CoV-2 has infected more than 600 million people, resulting in more than 6 million deaths in the world (https://coronavirus.jhu.edu/). A better understanding of virus-host interactions, particularly how SARS-CoV-2 interacts with the host immune system, will facilitate the development of new antiviral approaches and a better understanding of disease.

SARS-CoV-2 is a member of the *Betacoronavirus* genus within the *Coronaviridae* family containing a single-stranded positive-sense RNA genome that encodes four structural proteins [spike (S), membrane (M), envelope (E), and nucleocapsid (N)], 16 non-structural proteins (NSP1–NSP16), and seven accessory proteins (ORF3a, ORF3b, ORF6, ORF7a, ORF7b, ORF8, and ORF10) (3, 4). The accessory proteins of coronaviruses have been generally considered to be dispensable for viral replication but thought to play important roles in pathogenesis including antagonism of host innate immune responses (5 - 7).

The host innate immune system plays a critical role in controlling viral infection in both barrier epithelial cells and more specialized immune cells (8, 9). Upon entry into target cells, viral RNAs can be recognized by RIG-I-like receptors (RLRs) (10 - 12), which interact with adaptor protein MAVS, leading to the activation of TBK1 and the transcription factor IRF3. Phosphorylated IRF3 translocates into the nucleus and induces type I and type III interferons (IFNs) and other proinflammatory cytokines. IFNs bind to their receptors on respiratory cells to activate the JAK-STAT signaling pathway, leading to the phosphorylation of STAT1 and STAT2 and their nuclear translocation to stimulate the expression of hundreds of interferon-stimulated genes (ISGs) (13 - 15). Many of these ISGs execute antiviral activities.

For successful infection of hosts, viruses antagonize or evade this IFN pathway. Coronaviruses encode many accessory proteins, and some of these have been previously shown to antagonize IFN signaling. Indeed, in SARS-CoV-1, studies found that ORF6 inhibits the production of IFN and ISGs by disrupting IRF3 phosphorylation and translocation as well as STAT1 translocation (6, 7). Using overexpression systems, studies showed that ectopic expression of SARS-CoV-2 ORF6 can block IFN production and IFN signaling in non-respiratory cells (16 - 24). For example, overexpression of ORF6 blocks Sendai virus (SeV)- and Poly(I:C)-induced IFN and IFN-β-induced ISGs in HEK293T cells (17). Two studies compared the infection of WT SARS-CoV-2 and an ORF6-deleted virus (25, 26). In one study, the authors found that they exhibit similar replication kinetics in Vero cells, human HEK293T, and A549 cells expressing hACE2 receptor (25). By contrast, another study showed that the replication of ORF6-deleted SARS-CoV-2 was comparable in Vero and A549-ACE2 cells but attenuated at late time points in human A549-ACE2 cells (26). Moreover, *in vivo* studies in mice found no attenuation of infection comparing WT and ORF6-deleted SARS-CoV-2 (26). Therefore, the role of ORF6 in viral replication and the IFN response during SARS-CoV-2 infection in the respiratory tract remains unclear.

By comparing WT and ORF6-deleted (ΔORF6) SARS-CoV-2 viruses, we investigated the role of ORF6 in infection and IFN signaling in respiratory epithelial cells. Calu-3 cells are a human respiratory model that endogenously expresses the entry factors ACE2 and TMPRSS2, have intact innate immune signaling pathways, and are highly permissive to SARS-CoV-2 infection (27, 28). Surprisingly, compared to WT virus, we found that the ΔORF6 virus replicated to higher levels early in infection of human respiratory Calu-3 cells. This increased infection was associated with increased induction of type I and type III IFNs, ISGs, and proinflammatory cytokines in lung epithelial cells. We previously showed that IFNs and ISGs are induced in bystander cells (29), and thus, we monitored the activation of these pathways in WT and ΔORF6 virus-infected cells. These studies revealed that IRF3 and STAT1 translocation were only activated in bystander cells, suggesting that ORF6 is not required for the evasion of these pathways during infection. We next explored if exogenous activation of these pathways by potent ligands could be modulated by ORF6 expressed during *bona fide* SARS-CoV-2 infection. We observed no difference in SeV-induced IRF3 translocation between WT SARS-CoV-2 and ΔORF6-infected cells. And, the induction of IFNs was also unaffected by SARS-CoV-2 infection with either virus. Furthermore, we found that pretreatment of type I and type III IFNs restricted SARS-CoV-2 infection of both WT and ΔORF6 viruses and had no effect on IFN-β-stimulated ISG induction. However, we did observe that there was an increase in STAT1 translocation in IFN-β-stimulated SARS-CoV-2 ΔORF6-infected cells, suggesting antagonism under conditions of high stimulation. Taken together, our study suggests that ORF6 is not sufficient to antagonize IFN production or IFN signaling in SARS-CoV-2-infected respiratory cells but may impact efficacy IFNs delivered by professional immune cells or therapeutics that stimulate IFN signaling.

## RESULTS

### ΔORF6 virus efficiently replicates and induces immune responses in human respiratory epithelial cells

**Fig 1:**
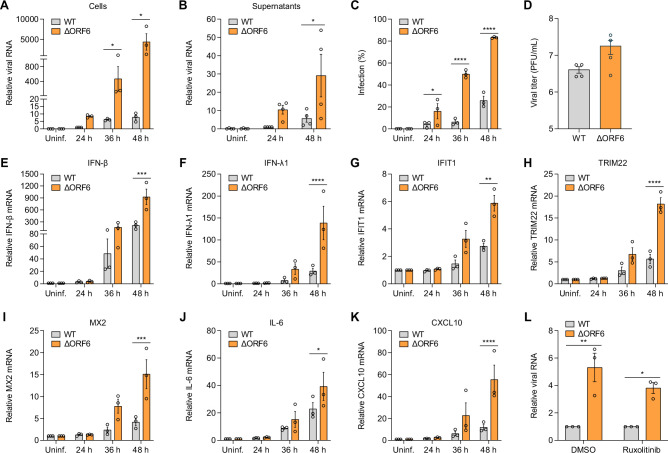
ORF6 mutant SARS-CoV-2 replicates efficiently in respiratory cells. (**A**) Calu-3 cells were either uninfected (Uninf.) or infected with SARS-CoV-2 WT virus or ΔORF6 virus (MOI of 0.5) for 24, 36, or 48 h. Viral replication was analyzed by RT-qPCR. Viral RNA of WT virus at 24 h was set to 1. Graphs show the means ± SEM for three independent experiments. (**B**) Calu-3 cells were either uninfected (Uninf.) or infected with SARS-CoV-2 WT virus or ΔORF6 virus (MOI of 0.5) for 4 h, and then inoculum was removed. Supernatants were collected at 24 or 48 h. Viral release into the supernatants was analyzed by RT-qPCR. Viral RNA of WT virus at 24 h was set to 1. Graphs show the means ± SEM for four independent experiments. (**C**) Percent of infection was determined by staining of SARS-CoV-2 Spike protein with automated microscopy. Graphs show the means ± SEM for three independent experiments. (**D**) SARS-CoV-2 WT virus or ΔORF6 virus was titered on Calu-3 cells. Shown are the means ± SEM for four independent experiments. (E through K) Calu-3 cells were either uninfected (Uninf.) or infected with SARS-CoV-2 WT virus or ΔORF6 virus (MOI of 0.5) for 24, 36, or 48 h. Total RNA was extracted, and the mRNA expression of IFN-β (**E**), IFN-λ1 (**F**), IFIT1 (**G**), TRIM22 (**H**), MX2 (**I**), IL-6 (**J**), CXCL10 (**K**) was examined by RT-qPCR. Gene expression (gene/18S) was normalized to uninfected cells. Shown are the means ± SEM for three independent experiments. (**L**) Calu-3 cells were pretreated with DMSO or 10 µM Ruxolitinib for 1 h, followed by infection of SARS-CoV-2 WT virus or ΔORF6 virus (MOI of 0.5). At 48 hpi, viral RNA was examined by RT-qPCR and normalized to WT virus-infected cells. Shown are the means ± SEM for three independent experiments. For all graphs, the significance was calculated using two-way analysis of variance (ANOVA) and is indicated by *P < 0.05, **P < 0.01, ***P < 0.001, and ****P < 0.0001. RT-qPCR primer information is shown in Table 1.

ORF6 proteins from both SARS-CoV-1 and SARS-CoV-2 have been implicated in immune evasion (6, 16, 17). Previous studies have also shown that ORF6 proteins from SARS-CoV-1 and SARS-CoV-2 are not required for viral replication *in vitro* or *in vivo* in mouse models (5, 25, 26). To determine the role of ORF6 in SARS CoV-2 infection in human respiratory epithelial cells, we infected Calu-3 cells with SARS-CoV-2 WT virus or ΔORF6 virus and monitored viral replication using RT-qPCR and automated microscopy at different time points post infection. We found that the ΔORF6 virus replicated more efficiently than WT virus, as evidenced by more viral RNA in the cells (Fig. 1A) and in the supernatants (Fig. 1B) at each time point. Using automated microscopy, we found a higher percentage of infection in ΔORF6 virus-infected cells (Fig. 1C). We also observed robust viral replication of ΔORF6 virus in human respiratory cell line A549 ectopically expressing SARS-CoV-2 receptor ACE2 (Fig. S1A). Next, we tittered these two viruses in both Calu-3 and Vero TMPRSS2 cells. Consistent with previous studies, ORF6 had only a modest effect on the infectivity of Calu-3 cells (Fig. 1D) and Vero TMPRSS2 cells (Fig. S1B), suggesting that the mutant replicated faster but to a similar extent in these cell types.

**TABLE 1:** Primers used in this study

Gene	Forward primer (5' to 3')	Reverse primer (5' to 3')
18S RNA	AACCCGTTGAACCCCATT	CCATCCAATCGGTAGTAGCG
IFN-β	GCTTCTCCACTACAGCTCTTTC	CAGTATTCAAGCCTCCCATTCA
IFN-λ1	ATCCTCTCCCAGCTCCAG	AGGTTGAAGGTGACAGATGC
IFIT1	CAACCAAGCAAATGTGAGGA	AGGGGAAGCAAAGAAAATGG
TRIM22	CTGTCCTGTGTGTCAGACCAG	TGTGGGCTCATCTTGACCTCT
MX2	CAGAGGCAGCGGAATCGTAA	TGAAGCTCTAGCTCGGTGTTC
IL-6	ACTCACCTCTTCAGAACGAATTG	CCATCTTTGGAAGGTTCAGGTTG
CXCL10	GTGGCATTCAAGGAGTACCTC	TGATGGCCTTCGATTCTGGATT
SARS-CoV-2 N gene	TTACAAACATTGGCCGCAAA	GCGCGACATTCCGAAGAA

We and others found that SARS-CoV-2 infection stimulates a delayed immune response in respiratory epithelial cells (17, 29). Indeed, we observed the induction of type I IFN (IFN-β), type III IFN (IFN-λ1), ISGs (IFIT1, TRIM22, and MX2), and proinflammatory cytokines and chemokines (IL-6 and CXCL10) at late time points upon viral infection by RT-qPCR (Fig. 1E through K). Consistent with robust replication of the ΔORF6 virus (Fig. 1A), we observed a stronger induction in ΔORF6 virus-infected cells compared to WT virus-infected cells (Fig. 1E through K).

Because both WT and ΔORF6 viruses can induce IFNs in respiratory epithelial cells, we set out to examine the role of IFNs produced during infection. Antiviral ISGs are induced by JAK-STAT signaling downstream of IFN receptors. We took advantage of the JAK inhibitor ruxolitinib to block IFN-dependent gene expression during infection (29, 30). As we previously found, ruxolitinib had a modest effect on infection of WT virus, and ΔORF6 virus was not differentially sensitive to JAK inhibition as measured by RT-qPCR (Fig. 1L). This suggests that SARS-CoV-2-induced IFN signaling pathways are not sufficient to block viral infection, likely due to delayed immune response induced by viral infection and that ORF6 does not play a role in this.

### WT and ΔORF6 virus induce IFN signaling in bystander cells

**Fig 2:**
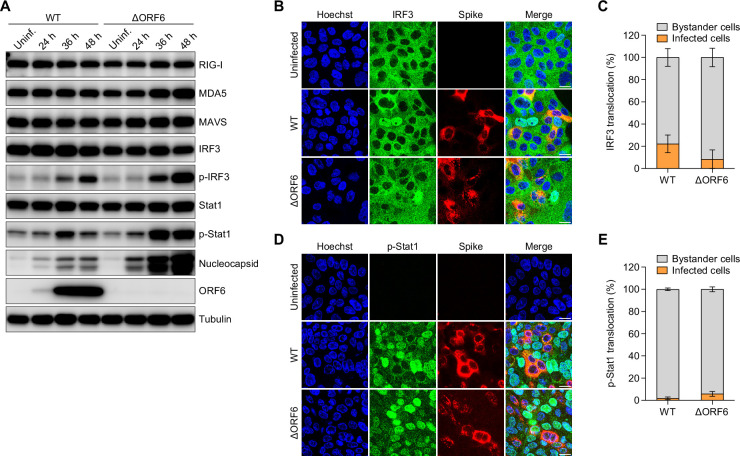
SARS-CoV-2 induces immune responses in bystander cells. (**A**) Calu-3 cells were either uninfected (Uninf.) or infected with SARS-CoV-2 WT virus or ΔORF6 virus (MOI = 0.5) for 24, 36, or 48 h. Cells were lysed, and the protein expression level was determined by immunoblotting using indicated antibodies. Shown are representative blots of two independent experiments. (**B through E**) Calu-3 cells were either uninfected or infected with SARS-CoV-2 WT virus or ΔORF6 virus (MOI of 0.5) for 48 h. Cells were fixed and stained with antibodies against IRF3 (**B**) or phospho-STAT1 (p-Stat1) (**D**). Quantification of IRF3 nuclear translocation (**C**) in panel (**B**) or p-Stat1 nuclear translocation (**E**) in panel (**D**). Graphs show the average percent of nuclear translocation in viral-infected cells or bystander cells for six fields of view collected from two independent experiments.

Viral recognition by RLRs activates a series of signaling cascades that initiate IFN induction and ISG production. To determine the role of ORF6 in the activation of immune pathways, we infected Calu-3 cells with WT or ΔORF6 SARS-CoV-2 and monitored the activation status of diverse innate sensing pathways by immunoblot. We examined the RLR RNA sensors RIG-I and MDA5, the adaptor protein MAVS, the transcription factor IRF3, and downstream ISG inducer STAT1 at 24, 36, and 48 hpi. We found that ΔORF6 virus exhibited a mild effect on the expression levels of RIG-I, MDA5, and MAVS, but that phosphorylated IRF3 and STAT1 were more strongly induced in ΔORF6 virus-infected cells than in WT virus-infected cells at late time points (Fig. 2A). This is in agreement with the higher levels of viral replication as we observed by immunoblot against Nucleocapsid (Fig. 2A) and qPCR (Fig. 1A), as well as our observed increased induction of IFNs and ISGs in ΔORF6 virus-infected cells (Fig. 1E through K).

If ORF6 was antagonizing these innate pathways directly, we should observe increased activation in infected cells. We have previously shown that SARS-CoV-2 elicits innate signaling in bystander cells: IRF3 translocation and TBK1 activation exclusively induced in neighboring cells (29). To investigate a possible role of ORF6 in antagonizing activation in infected cells, we infected cells with WT or ΔORF6 virus for 48 h and monitored IRF3 nuclear translocation and viral infection at single-cell resolution by confocal microscopy. As expected, WT virus-induced IRF3 translocation occurred in neighboring uninfected cells as measured by the lack of cells co-staining with IRF3 and SARS-CoV-2 Spike protein. Similar to WT virus, we found that ΔORF6-induced IRF3 nuclear translocation was primarily in bystander cells (Fig. 2B and C). To examine downstream IFN signaling at the single-cell level, we examined STAT1 activation by confocal microscopy. We found that both WT virus and ΔORF6 virus stimulated STAT1 activation (phospho-STAT1) predominantly in uninfected bystander cells (Fig. 2D and E). Therefore, loss of ORF6 did not impact IFN or ISG induction in infected cells.

### WT and ΔORF6 virus are similarly sensitive to restriction by IFNs

**Fig 3:**
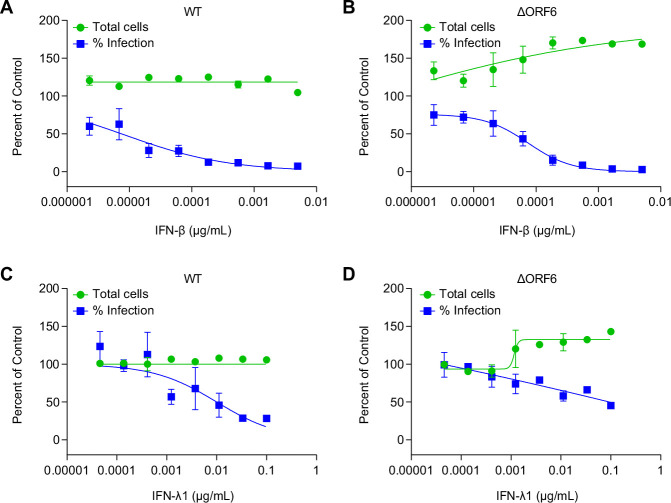
IFNs block SARS-CoV-2 infection. Calu-3 cells were treated with IFN-β (**A and B**) and IFN-λ1 (**C and D**) at the indicated concentrations and subsequently infected with SARS-CoV-2 WT virus or ΔORF6 virus (MOI of 0.5). At 48 hpi, cells were stained for dsRNA (anti-J2) and imaged by automated microscopy. Percentage of viral infection and cell viability were quantified and normalized to vehicle control.

While paradoxically ΔORF6 virus replicated more efficiently in respiratory epithelial cells than WT virus even in the presence of higher ISG expression, we next tested if the two viruses were similarly sensitive to IFN pretreatment. We and others have shown that pretreatment with IFNs inhibits SARS-CoV-2 infection (31 - 33). We tested whether pretreatment with type I IFN (IFN-β) or type III IFN (IFN-λ1) would potentially be more active against ΔORF6 virus infection. We pretreated Calu-3 cells with IFNs at different concentrations for 1 h and then infected cells with either WT or ΔORF6 virus for 48 h. We quantified viral infection and cell viability using automated microscopy. We found that IFN-β potently blocked both WT virus and ΔORF6 virus infection in a dose-dependent manner, and their sensitivity to the antiviral activity of IFN-β was comparable (Fig. 3A and B). Compared to IFN-β, IFN-λ1 was less active, even at high concentrations, but similarly blocked both viral infections (Fig. 3C and D).

### ORF6 does not interfere with IFN production

**Fig 4:**
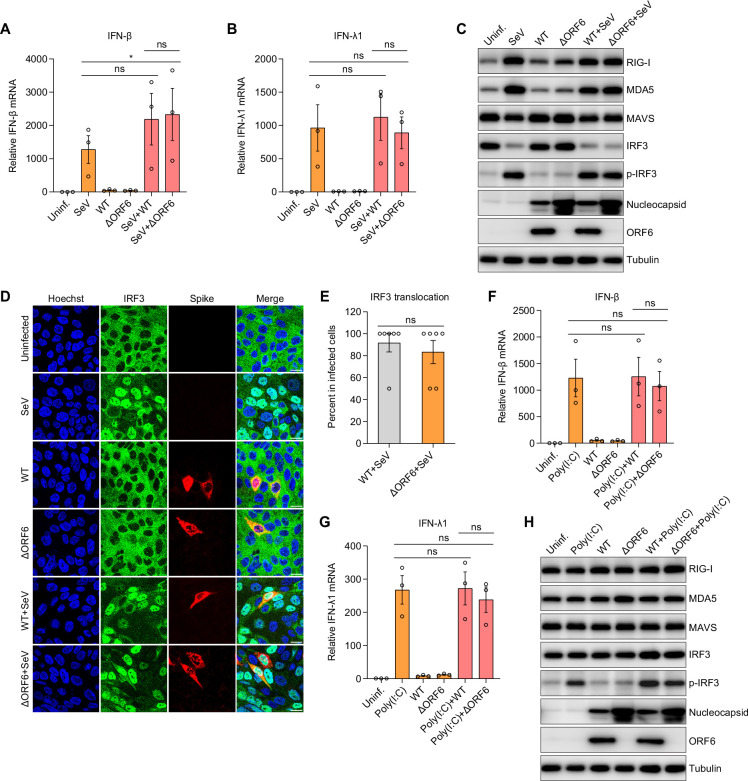
ORF6 does not antagonize IFN production. (A–E) Calu-3 cells were uninfected or infected with SARS-CoV-2 WT virus or ΔORF6 virus (MOI of 0.5). At 24 hpi, cells were treated with Sendai virus (SeV) for 8 h as indicated. (**A and B**) Total RNA was extracted, and induction of IFN-β (**A**) and IFN-λ1 (**B**) was examined by RT-qPCR. Shown is the mean ± SEM for three independent experiments. The significance was calculated using Kruskal-Wallis test and is indicated by *P < 0.05 (ns, not significant). (**C**) Cell lysates were collected, and the protein expression level was determined by immunoblotting using indicated antibodies. Shown are the representative blots of two independent experiments. (**D**) Cells were fixed and stained with antibodies against IRF3 and SARS-CoV-2 Spike. Representative images of two independent experiments are shown. (**E**) Percent of IRF3 translocation in virally infected cells was quantified. Shown is the average percent for six fields of view from two independent experiments. Significance was calculated using an unpaired, two-tailed Student’s *t* test (ns, not significant). (F through H) Calu-3 cells were infected with SARS-CoV-2 WT virus or ΔORF6 virus (MOI of 0.5) for 24 h, followed by treatment of Poly(I:C) for 6 h as indicated. (**F and G**) mRNA expression of IFN-β (**F**) and IFN-λ1 (**G**) was determined by RT-qPCR and normalized to uninfected cells. Shown are the means ± SEM for three independent experiments. The significance was calculated using Kruskal-Wallis test (ns, not significant). (**H**) Immunoblotting was performed to monitor protein expression using indicated antibodies.

Previous studies showed that ectopic expression of SARS-CoV-2 ORF6 blocks SeV-induced IFN-β promoter activity in HEK293T cells (17). The levels of ORF6 expression and binding partners are likely different during *bona fide* infection of respiratory cells. We next determined the role ORF6 in IFN induction in the context of exogenous stimulation. To this end, we infected Calu-3 cells with SARS-CoV-2 WT virus or ΔORF6 virus for 24 h and then treated these cells with SeV for 8 h. We examined the expression of type I IFN (IFN-β) and type III IFN (IFN-λ1) mRNAs. As expected, SeV alone potently stimulated IFN production at 8 h. Consistent with the results shown in Fig. 1, WT virus or ΔORF6 virus-infected cells showed little induction of IFNs at 24 hpi. If SARS-CoV-2 ORF6 antagonizes SeV-induced IFN production, we expected that the SeV-induced IFN induction would be reduced in WT SARS-CoV-2-infected cells compared to ΔORF6-infected cells, which we did not observe (Fig. 4A and B). Moreover, compared to SeV-treated cells, we observed no decrease in IFNs by WT or ΔORF6 SARS-CoV-2 infections (Fig. 4A and B).

Next, we monitored the activation status of the canonical RLR sensing pathways upon treatment of SeV in the presence or absence of SARS-CoV-2 infection by immunoblot (Fig. 4C). We found that SeV treatment alone upregulated RIG-I and MDA-5, along with increased phosphorylated IRF3 at 8 hpi. In contrast, SARS-CoV-2 infection alone, either WT or ΔORF6, had no effect on these proteins at 24 hpi while we did observe increased nucleocapsid production during infection with ΔORF6 in the presence or absence of SeV. Moreover, SeV pretreatment did not impact the level of infection of either virus. Next, we examined the IRF3 activation when we coinfected cells with SeV and WT or ΔORF6 SARS-CoV-2. Compared to SeV alone, we observed no difference of total IRF3 or phosphorylated IRF3 in SeV and SARS-CoV-2, either WT virus or ΔORF6 virus co-infected cells (Fig. 4C). We also observed no differences in the levels of RIG-I or MDA5 between SeV alone and SeV co-infections with either WT or ΔORF6 SARS-CoV-2 (Fig. 4C).

Since previous studies showed that overexpression of ORF6 blocks IRF3 translocation upon SeV treatment (17), we further examined this at single-cell resolution using confocal microscopy. Calu-3 cells were infected with WT or ΔORF6 SARS-CoV-2 for 24 h, followed by treatment with SeV for 8 h. We found that while SeV strongly induced IRF3 translocation, in contrast, infection with either WT or ΔORF6 SARS-CoV-2 virus did not induce IRF3 activation at this early time point 24 hpi. Moreover, we found that SeV-induced IRF3 nuclear accumulation occurred to similar extents in co-infected cells with either WT virus or ΔORF6 virus (Fig. 4D and E). In addition to SeV, we treated cells with another potent IFN inducer Poly(I:C) and determined whether ORF6 could attenuate Poly(I:C)-induced IFN signaling. Again, we observed no significant difference in Poly(I:C)-induced IFN production during co-infection (Fig. 4F and G). Immunoblot analysis revealed that while infection with ΔORF6 SARS-CoV-2 was higher than WT SARS-CoV-2, its replication was insensitive to Poly(I:C). Again, SARS-CoV-2 infection with either WT or ΔORF6 had no impact on Poly(I:C)-induced IRF3 phosphorylation (Fig. 4H). Altogether, these data suggest that ORF6 does not antagonize stimuli-induced IFN production during SARS-CoV-2 infection.

### ORF6 blocks IFN-β-induced STAT1 nuclear translocation in infected cells but does not block IFN signaling

**Fig 5:**
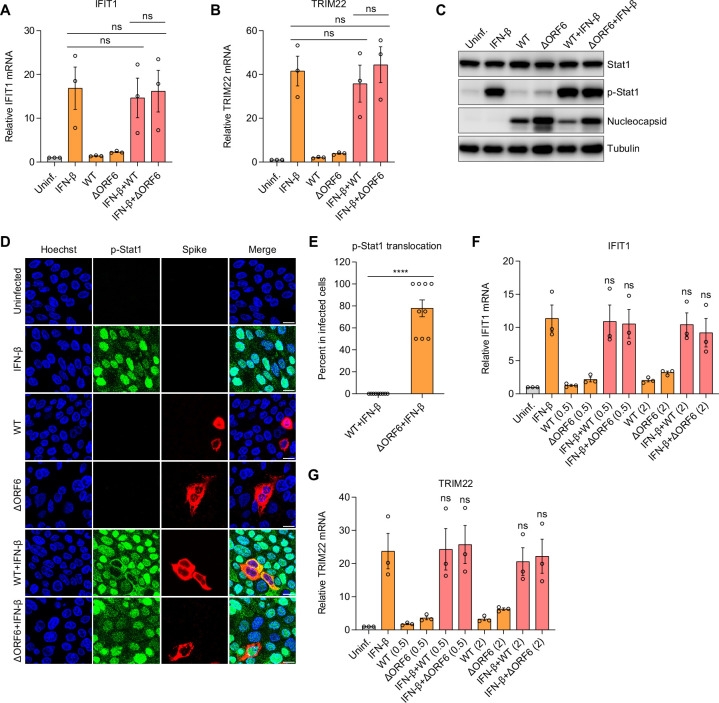
ORF6 attenuates IFN-stimulated Stat1 translocation but does not impact ISG production. (**A and B**) Calu-3 cells were infected with SARS-CoV-2 WT virus or ΔORF6 virus (MOI of 0.5) for 24 h. Cells were subsequently treated with IFN-β for 8 h as indicated. Induction of IFIT1 (**A**) and TRIM22 (**B**) was examined by RT-qPCR. Gene expression was normalized to uninfected cells. Shown is the mean ± SEM for three independent experiments. The significance was calculated using Kruskal-Wallis test (ns, not significant). (C–E) Calu-3 cells were infected with SARS-CoV-2 WT virus or ΔORF6 virus (MOI of 0.5). At 24 hpi, cells were treated with IFN-β for 0.5 h as indicated. (**C**) Cells were lysed, and the expression of total and phosphorylated STAT1 (p-Stat1) was determined by immunoblotting. Shown are the representative blots of two independent experiments. (**D**) Cells were fixed and stained with antibodies against p-Stat1 and SARS-CoV-2 Spike. Representative images of two independent experiments are shown. (**E**) Percent of p-Stat1 translocation in SARS-CoV-2 infected cells in panel D was determined. Shown is the average percent for nine fields of view from three independent experiments. Significance was calculated using an unpaired, two-tailed Student’s *t* test (****P < 0.0001). (**F and G**) Calu-3 cells were infected with SARS-CoV-2 WT virus or ΔORF6 virus with two MOI (0.5 and 2) for 24 h, followed by treatment of IFN-β for 8 h as indicated. mRNA expression of IFIT1 (**F**) and TRIM22 (**G**) was analyzed by RT-qPCR. Shown is the mean ± SEM for three independent experiments. The significance was calculated using Kruskal-Wallis test (ns, not significant).

Overexpression of SARS-CoV-2 ORF6 was found to block IFN signaling, as evidenced by decreased ISRE promoter activity in ORF6 expressing HEK293T cells upon IFN treatment (16, 17). We found that during infection of respiratory cells with either WT or ΔORF6 SARS-CoV-2, STAT1 activation occurs exclusively in bystander cells (Fig. 2B through E). We next tested whether SARS-CoV-2 infection impacts IFN-induced STAT1 activation and ISG production. We infected Calu-3 cells with WT or ΔORF6 SARS-CoV-2 for 24 h and then treated with IFN-β for 8 h. Induction of ISGs including IFIT1 and TRIM22 was measured by RT-qPCR. We found that ISGs were induced in IFN-β-treated cells but not in WT or ΔORF6 SARS-CoV-2-infected cells at 24 hpi (Fig. 5A and B). If SARS-CoV-2 ORF6 blocks IFN signaling, we expected a decreased level of ISGs in SARS-CoV-2 WT virus-infected cells but not in ΔORF6 virus-infected cells. By contrast, we observed a comparable level of IFN-β-stimulated ISG expression between control and WT or ΔORF6 SARS-CoV-2-infected cells (Fig. 5A and B).

Next, we monitored the IFN-β-induced STAT1 activation with or without SARS-CoV-2 infection by immunoblot. We observed a similar level of STAT1 phosphorylation in IFN-β-treated cells in the absence or presence of SARS-CoV-2 infection with either virus, suggesting that ORF6 does not block STAT1 phosphorylation (Fig. 5C). Phosphorylated STAT1 and STAT2 translocate into the nucleus to stimulate ISG transcription. Previous studies showed that ectopic expression of ORF6 inhibits STAT1 nuclear translocation (16, 17, 22). We tested this by infecting Calu-3 cells with WT or ΔORF6 SARS-CoV-2 for 24 h and then treating cells with IFN-β for 30 min. We monitored STAT1 nuclear translocation by confocal microscopy. Upon IFN-β treatment alone, we observed an efficient translocation of STAT1 into the nucleus. Moreover, there was no translocation of STAT1 upon infection with WT or ΔORF6 SARS-CoV-2 at 24 hpi. We found that IFN-β-induced STAT1 translocation was blocked in SARS-CoV-2 WT virus-infected cells but not in ΔORF6 virus-infected cells, suggesting that ORF6 can suppress STAT1 nuclear translocation when induced at high levels by exogenous IFN-β (Fig. 5D and E).

However, reduced translocation of STAT1 in SARS-CoV-2 WT virus-infected cells would predict a corresponding decrease in IFN-stimulated ISG induction. We observed no significant difference in ISGs induction upon IFN-β treatment, whether the cells were infected with WT or ΔORF6 SARS-CoV-2. This could be due to low levels of SARS-CoV-2 infection. To test this hypothesis, we infected cells with increasing doses of SARS-CoV-2 and examined the effect of viral infection on IFN signaling and ISG production. Again, we observed no difference in IFN-β-induced ISG expression in uninfected or viral-infected cells even at high MOIs we tested (Fig. 5F and G). Collectively, these data suggest that ORF6 is not sufficient to antagonize IFN signaling during infection but can attenuate STAT1 translocation upon exogenous stimulation with IFN-β with little impact on ISG expression.

## DISCUSSION

As the first and most rapid line of defense against invading viral pathogens, the activation of innate immune signaling in infected barrier cells plays an important role in the restriction of viral replication and spread (34 - 37). The production of cytokines and chemokines induced by barrier cells leads to the recruitment of innate immune cells which can also produce IFNs and subsequently induce adaptive immune responses (38, 39). To establish infection, viruses must evade early innate immune responses including antiviral IFN pathways. Indeed, SARS-CoV-2 has been suggested to use diverse strategies to antagonize IFN responses. Ectopic expression strategies demonstrate that a given viral protein “can” impact IFN signaling, and using such a strategy, several SARS-CoV-2 proteins were found to block the IFN pathway at different steps including NSP1, NSP3, NSP6, NSP12, NSP13, NSP14, ORF3a, ORF6, and ORF7 (16, 17, 21). Among them, ectopic expression of ORF6 in HEK293T cells was shown to suppress both IFN production and IFN signaling by interfering with IRF3 and STAT1 nuclear translocation (16, 17, 22). ORF6 from SARS-CoV-1 was also found to block IFN responses by disrupting IRF3 phosphorylation and translocation as well as STAT1 translocation (6, 7). SARS-CoV-1 ORF6 is dispensable for viral replication *in vitro* and *in vivo* (5, 40). The previous studies showed that compared to SARS-CoV-1 WT virus, the replication of recombinant ΔORF6 virus remained similar or was slightly decreased in cell cultures and mice (5, 41). Using hACE2 transgenic K18 mice (K18-hACE2), a recent study found that ORF6-deleted SARS-CoV-2 showed similar weight loss and viral titers to WT virus *in vivo* (26).

In this study, we set out to determine the role of ORF6 in viral infection and IFN responses during SARS-CoV-2 infection in respiratory epithelial cells. Interestingly, we found that SARS-CoV-2 ΔORF6 virus-infected cells exhibited more rapid infection of respiratory cells. As expected, increased replication led to increased IFNs, ISGs, and proinflammatory cytokines in Calu-3 cells. Likewise, we observed stronger induction of phosphorylated IRF3 and STAT1 in ΔORF6 virus-infected cells. This increased replication was independent of IFN signaling as treatment with the JAK inhibitor ruxolitinib did not impact the levels of infection differentially between WT and ΔORF6 SARS-CoV-2.

We previously showed that SARS-CoV-2-dependent IFN responses occurred in uninfected bystander cells (29). We explored whether this was dependent on ORF6. However, we found that ΔORF6 virus-induced IRF3 and STAT1 nuclear translocation occurred in neighboring uninfected cells but not in virus-infected cells. This demonstrates that the evasion of these pathways during infection is ORF6-independent. Next, we explored how ORF6 produced during SARS-CoV-2 infection could impact the exogenous activation of IFN or ISGs. Previous studies showed that overexpression of SARS-CoV-2 ORF6 in HEK293T cells interferes with IRF3 translocation into the nucleus and inhibits IFN production upon SeV treatment (16, 17). By contrast, we found that SeV-induced IRF3 nuclear translocation occurred to a similar extent in SeV-treated cells that were uninfected or infected with WT or ΔORF6 SARS-CoV-2 and that IFN production remained induced to the same level as SeV treatment alone. This suggests that ORF6 does not antagonize IRF3 translocation or IFN induction in respiratory cells when expressed by SARS-CoV-2.

Ectopic expression of ORF6 from SARS-CoV-1 or SARS-CoV-2 has demonstrated that ORF6 can physically interact with the Nup98-Rae1 nuclear pore complex and disrupt nuclear import of STAT1, resulting in decreased ISGs induction in IFN-β-treated cells (22, 23, 42). While ORF6 did not block STAT1 translocation during SARS-CoV-2 infection, we found that IFN-β-induced STAT1 translocation occurred in ΔORF6 SARS-CoV-2 virus-infected cells but not in WT SARS-CoV-2 virus-infected cells. These data suggest that ORF6 is able to block STAT1 nuclear translocation upon exogenous IFN treatment which may be due to other viral proteins that present in both WT and ΔORF6 SARS-CoV-2 can block STAT1 translocation only during a weak stimulus. Indeed, SARS-CoV-2 N and NSP13 have been shown to suppress STAT1 translocation into the nucleus (43, 44). The inhibitory effect of these additional viral proteins on STAT1 translocation can only be rescued by high concentration of artificial IFN treatment but not by IFN induced by viral infection. However, this had no observable impact on ISG production as comparable levels of ISGs were observed in IFN-β-treated cells in the presence or absence of SARS-CoV-2 infection with either WT or ΔORF6 virus. IFN responsed have been linked to disease severity in COVID-19 patients. Early IFN responses can decrease viral load and lead to mild COVID-19 symptoms (45). Interestingly, SARS-CoV-2 ORF6 deletion variants in patient samples exhibited similar replication kinetics as we observed in Calu-3 cells and antiviral ISG profiling as WT virus (46). Importantly, no difference in disease severity was observed between WT and ORF6 deletion variants-infected patients (46). These data argue against the role of ORF6 in antagonizing early IFN response, therefore impacting disease progression in COVID-19 patients. These data are consistent with our findings.

These data suggest that although ORF6 interferes with STAT1 nuclear translocation upon exogenous IFN treatment, STAT1 activation in uninfected bystander cells remains sufficient to induce ISGs. This is consistent with other studies demonstrating an uncoupling of these activities. For example, a recent study showed that upon IFN-α treatment, although SARS-CoV-2 infection reduces STAT1 phosphorylation, the STAT1 activation was sufficient to induce ISGs to the similar level of cells without infection (21). Again, consistent with these observations, pretreatment with either type I IFN (IFN-β) or type III IFN (IFN-λ1) blocked WT or ΔORF6 SARS-CoV-2 similarly (31 - 33). Together, these data suggest that virus-induced IFN from barrier cells is unable to block infection locally. This is likely due, at least in part, to the delayed induction of innate immune signaling pathways upon SARS-CoV-2 infection. Whether the recruitment of professional myeloid cells and their activation by SARS-CoV-2 antigens can induce higher levels of IFNs or therapeutics that induce antiviral IFN pathways would be attenuated by ORF6 *in vivo* needs to be further investigated.

## MATERIALS AND METHODS

### Cells and viruses

Calu-3 cells (HTB-55) were obtained from the American Type Culture Collection and were cultured in minimum essential medium, supplemented with 10% fetal bovine serum, 1% penicillin/streptomycin, 1% GlutaMAX (Gibco), and 1% non-essential amino acids 37°C and 5% CO2. A549-ACE2 cells were cultured in RPMI-1640 medium, supplemented 10% fetal bovine serum and 1% penicillin/streptomycin.

SARS-CoV-2 WT virus and SARS-CoV-2 ΔORF6 viruses were previously described (26). Work with SARS-CoV-2 infection was performed in a biosafety level three laboratory and approved by the Institutional Biosafety Committee and Environmental Health and Safety.

### Reagents and antibodies

IFN-β and IFN-λ1 were purchased from BioLegend. SeV was purchased from Charles River, and Poly(I:C) was purchased from Invivogen. Ruxolitinib was purchased from Selleck Chemicals. The following antibodies were used for Western blotting or immunofluorescence: anti-RIG-I (Cell Signaling Technology, 3743S), anti-MDA-5 (Cell Signaling Technology, 5321S), anti-MAVS (Cell Signaling Technology, 3993S), anti-IRF3 (Cell Signaling Technology, 11904S), anti-Phospho-IRF3 (Ser396) (Cell Signaling Technology, 4947S), STAT1 (Cell Signaling Technology, 14994), p-STAT1 (Cell Signaling Technology, 9167), anti-SARS-CoV-2 nucleocapsid (GeneTex, GTX135357), anti-SARS-CoV-2 ORF6 (Novus Biologicals, NBP3-05707), anti-SARS-CoV-2 Spike (Absolute Antibody, CR3022), and anti-Tubulin (Sigma, T6199). HRP-conjugated secondary antibodies (anti-mouse or anti-rabbit) were purchased from Amersham. Alexa Fluor fluorescent secondary antibodies were purchased from Invitrogen.

### Dose-response studies and titers

For dose-response studies Calu-3 cells (7.5 × 10^3^ cells per well) were seeded in 384-well plates coated with collagen I (Corning). The next day, Calu-3 cells were treated with IFN-β or IFN-λ1 for 2 h at the indicated concentrations and subsequently infected with SARS-CoV-2 wild-type or ΔORF6 viruses (MOI of 0.5). At 48 hpi, cells were fixed with 4% formaldehyde for 15 min and then washed three times with PBS. Cells were blocked with 2% BSA in PBS with 0.1% Triton X-100 (PBST) for 1 h and incubated with anti-dsRNA (J2) antibody overnight at 4°C. Calu-3 cells were washed three times with PBST and then incubated with Hoechst 33342 (Sigma) and Alexa Fluor 488 conjugated anti-mouse secondary antibody for 1 h at room temperature. Cells were washed three times with PBST and imaged using an ImageXpress Micro 4 High-Content Imaging System (Molecular Devices) at 10× magnification. The cell number and the percentage of infected cells were quantified by MetaXpress software. Viral infection was normalized to DMSO control and calculated as Percent of Control (% Infection_sample_ / Average % Infection_DMSO_) × 100). For titers, Calu-3 cells (4.5 × 10^4^) or Vero TMPRSS2 cells (2.5 × 10^4^) were plated in 96 well plates; the next day, serial 10-fold dilutions of SARS-CoV-2 WT virus or ΔORF6 were used to infect the cells. At 48 hpi, cells were fixed with 4% formaldehyde for 15 min and then washed three times with PBS. Cells were blocked with 2% BSA in PBS with 0.1% Triton X-100 (PBST) for 1 h and incubated with anti-dsRNA (J2) antibody overnight at 4°C. Cells were washed three times with PBST and then incubated with Hoechst 33342 (Sigma) and Alexa Fluor 488 conjugated anti-mouse secondary antibody for 1 h at room temperature. Cells were washed three times with PBST and imaged using an ImageXpress Micro 4 High-Content Imaging System (Molecular Devices) at 10× magnification. Titers were calculated using the Reed-Muench method (47).

### RNA isolation and RT-qPCR

Calu-3 cells (7 × 10^5^ cells per well), A549-ACE2 cells (7.5 × 10^5^ cells per well) were seeded into 6-well plates. The next day, cells were incubated with SARS-CoV-2 WT virus or SARS-CoV-2 ΔORF6 virus for the indicated time point. Total RNA was extracted using TRIzol Reagent (Invitrogen) and purified using RNA Clean & Concentrator Kits (Zymo Research). cDNA was synthesized from the RNA samples with M-MLV Reverse Transcriptase (Invitrogen) and random hexamer primers. A 25-fold dilution of cDNA samples and Power SYBR Green PCR Master Mix (Applied Biosystems) were used for RT-qPCR analysis using QuantStudio 6 Flex Real-Time PCR Systems (Applied Biosystems). The expression levels of target genes were calculated using the standard curve method and normalized to 18S ribosomal RNA.

### Western blotting

Calu-3 cells (7 × 10^5^ cells per well) seeded into 6-well collagen-coated plates were infected with SARS-CoV-2 WT virus or SARS-CoV-2 ΔORF6 virus for 24, 36, or 48 h. To determine the role of SARS-CoV-2 in IFN production and IFN signaling, Calu-3 cells were incubated with SARS-CoV-2 WT virus or SARS-CoV-2 ΔORF6 virus for 24 h and subsequently treated with SeV, Poly(I:C) or IFN-β. At the indicated time points, Calu-3 cells were washed with PBS and lysed in RIPA buffer (50 mM Tris-HCl, 150 mM NaCl, 0.5% sodium deoxycholate, 0.1% SDS, 1% NP-40) supplemented with protease inhibitor cocktail (Sigma). Cell lysates were clarified at 13,000 rpm for 10 min at 4°C and incubated with 6× sample buffer at 95°C for 10 min. Proteins were separated on 12% SDS-PAGE gels and detected by using the indicated antibodies.

### Confocal microscopy

Calu-3 cells (1.5 × 10^5^ cells per well) were seeded on collagen-coated glass coverslips (Electron Microscopy Sciences). After 24 h, cells were infected with SARS-CoV-2 for 48 h or infected with SARS-CoV-2 for 24 h and then treated with SeV or IFN-β. Calu-3 cells were fixed with 4% formaldehyde for 10 min at room temperature and then blocked with 2% BSA in PBS with 0.1% Triton X-100 (PBST) for 1 h. Cells were incubated with the indicated primary antibodies overnight at 4°C. Coverslips were washed three times with PBST and then incubated with Alexa Fluor fluorescent secondary antibodies and Hoechst 33342 (Sigma) for 1 h at room temperature. Coverslips were washed three times with PBST, and cells were imaged using a Leica DM5500Q confocal microscope.

## SUPPLEMENTAL MATERIAL

Figure S1 mbio.01194-23-s0001.pdf SARS-CoV-2 and ORF6.

Supplemental figure legend mbio.01194-23-s0002.pdf Figure S1 legend.

ASM does not own the copyrights to Supplemental Material that may be linked to, or accessed through, an article. The authors have granted ASM a non-exclusive, world-wide license to publish the Supplemental Material files. Please contact the corresponding author directly for reuse.
