# Supplementary figures and images for: SARS-CoV-2 ORF6 protein does not antagonize interferon signaling in respiratory epithelial Calu-3 cells during infection
Source: mBio. 2023 Jun 28;14(4):e01194-23. doi: 10.1128/mbio.01194-23 (PMC10470815; doi:10.1128/mbio.01194-23)

**A**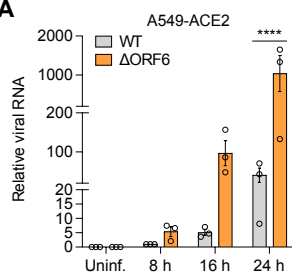**B**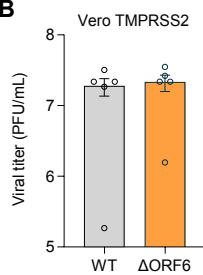

Supplement: Figure S1 — SARS-CoV-2 and ORF6. [file mbio.01194-23-s0001.pdf]
