# Supplementary material for: SARS-CoV-2 ORF6 protein does not antagonize interferon signaling in respiratory epithelial Calu-3 cells during infection
Source: mBio. 2023 Jun 28;14(4):e01194-23. doi: 10.1128/mbio.01194-23 (PMC10470815; doi:10.1128/mbio.01194-23)
Supplement: Supplemental figure legend — Figure S1 legend. [file mbio.01194-23-s0002.pdf]

FIG S1 Effect of ORF6 on SARS-CoV-2 infection. (A) A549-ACE2 cells were either uninfected (Uninf.) or infected with SARS-CoV-2 WT virus or ΔORF6 virus (MOI of 0.5) for 8, 16 or 24 hours. Viral RNA was analyzed by RT-qPCR. Viral RNA of WT virus at 8 hours was set to 1. Means ± SEM for three independent experiments shown. Significance was calculated using two-way analysis of variance (ANOVA) and is indicated by ****$P < 0.0001$. (B) SARS-CoV-2 WT virus or ΔORF6 virus was titered on Vero TMPRSS2 cells. Shown is the means ± SEM for four independent experiments.
